# An extremely rare case of desmoplastic fibroblastoma exhibiting rapid growth in the chest wall: a case report

**DOI:** 10.1186/s40792-021-01171-1

**Published:** 2021-04-07

**Authors:** Hideki Ota, Hirotaka Ishida, Hidekazu Matsumoto, Tomoharu Ishiyama

**Affiliations:** Department of Surgery, Yamagata Prefecture Shinjo Hospital, 12-55 Wakaba Town, Shinjo, Yamagata 996-0025 Japan

**Keywords:** Desmoplastic fibroblastoma, Collagenous fibroma, Chest wall tumor, Soft tissue, Rim enhancement, Videoscope

## Abstract

**Background:**

Desmoplastic fibroblastoma is an uncommon, benign, fibrous tumor exhibiting infiltrative growth. Most of these tumors are small, slow-growing, and develop as subcutaneous lesions in the extremities. Cases of desmoplastic fibroblastoma in the chest wall are quite rare, and the preoperative diagnosis of such cases remains challenging as these tumors can mimic the characteristics of desmoid-type fibromatosis, which often occurs in the chest wall. We aimed to describe a rare case of desmoplastic fibroblastoma exhibiting rapid growth in the chest wall of a patient that was successfully treated with marginal excision only by diagnostic imaging before surgery.

**Case presentation:**

A 79-year-old man was admitted to our hospital after experiencing right shoulder pain lasting for a few months. A 4 × 4 × 2 cm mass was incidentally detected at the right second rib two years prior. Chest computed tomography revealed a well-defined homogeneous mass with a muscle-like density along the right lateral chest wall, the size of which had increased to 12 × 10 × 4.5 cm in two years. Dynamic contrast-enhanced computed tomography revealed abundant vascularity at the periphery of the tumor. Magnetic resonance imaging revealed iso-intensity to muscle on T1-weighted images, slightly high intensity on T2-weighted images, and rim-like contrast enhancement at the periphery of the tumor, with uniform thickness on gadolinium-enhanced T1-weighted images with fat suppression. Rim-like contrast enhancement is an imaging feature that can distinguish cases of desmoplastic fibroblastoma from desmoid-type fibromatosis. We diagnosed the tumor as desmoplastic fibroblastoma by diagnostic imaging without tissue biopsy. Marginal excision with videoscopic assistance was performed through a small incision. The pathological diagnosis was desmoplastic fibroblastoma. The patient’s postoperative course was uneventful, and his shoulder pain was relieved after the surgery.

**Conclusions:**

Desmoplastic fibroblastoma in the chest wall is extremely rare, but should be considered in the differential diagnosis when desmoid-type fibromatosis is clinically suspected. Gadolinium-enhanced magnetic resonance imaging is helpful in confirming the differential diagnosis.

## Background

Desmoplastic fibroblastoma is an uncommon, benign, fibrous, soft tissue tumor exhibiting infiltrative growth [1–4]. These types of tumors occur mostly in the subcutaneous and skeletal muscle tissues of the extremities [1–3], and patients typically present with a history of a painless, slow-growing mass, often over a relatively long duration of time [1–3]. The diameter of these tumors typically ranges from 1 to 20 cm, with a median diameter of 3 cm [1, 2]. In general, treatment involves the marginal excision of the tumor, and there has been no reported incidence of local recurrence or metastasis [3, 5].

Desmoplastic fibroblastomas share some features with other infiltrative tumors with fibrous components [3–7]; these other tumors are treated via surgical resection with a wide margin and exhibit high rates of local recurrence and metastasis [8]. Therefore, recognizing the unique characteristics of desmoplastic fibroblastoma is clinically important for the differential diagnosis of these tumors [3–7], although precise preoperative diagnosis remains challenging, mainly owing to the lack of established imaging features distinct to desmoplastic fibroblastoma [5–7].

Cases of desmoplastic fibroblastoma in the chest wall are quite rare, and it is important to differentiate these tumors from cases of extra-abdominal desmoid-type fibromatosis, which often occur in the chest wall [8–10]. Here, we aimed to present a rare case of desmoplastic fibroblastoma exhibiting rapid growth in the chest wall, which was successfully treated with marginal excision only by diagnostic imaging before surgery. Gadolinium-enhanced magnetic resonance imaging (MRI) can help to distinguish this tumor from desmoid-type fibromatosis.

## Case presentation

**Fig. 1:**
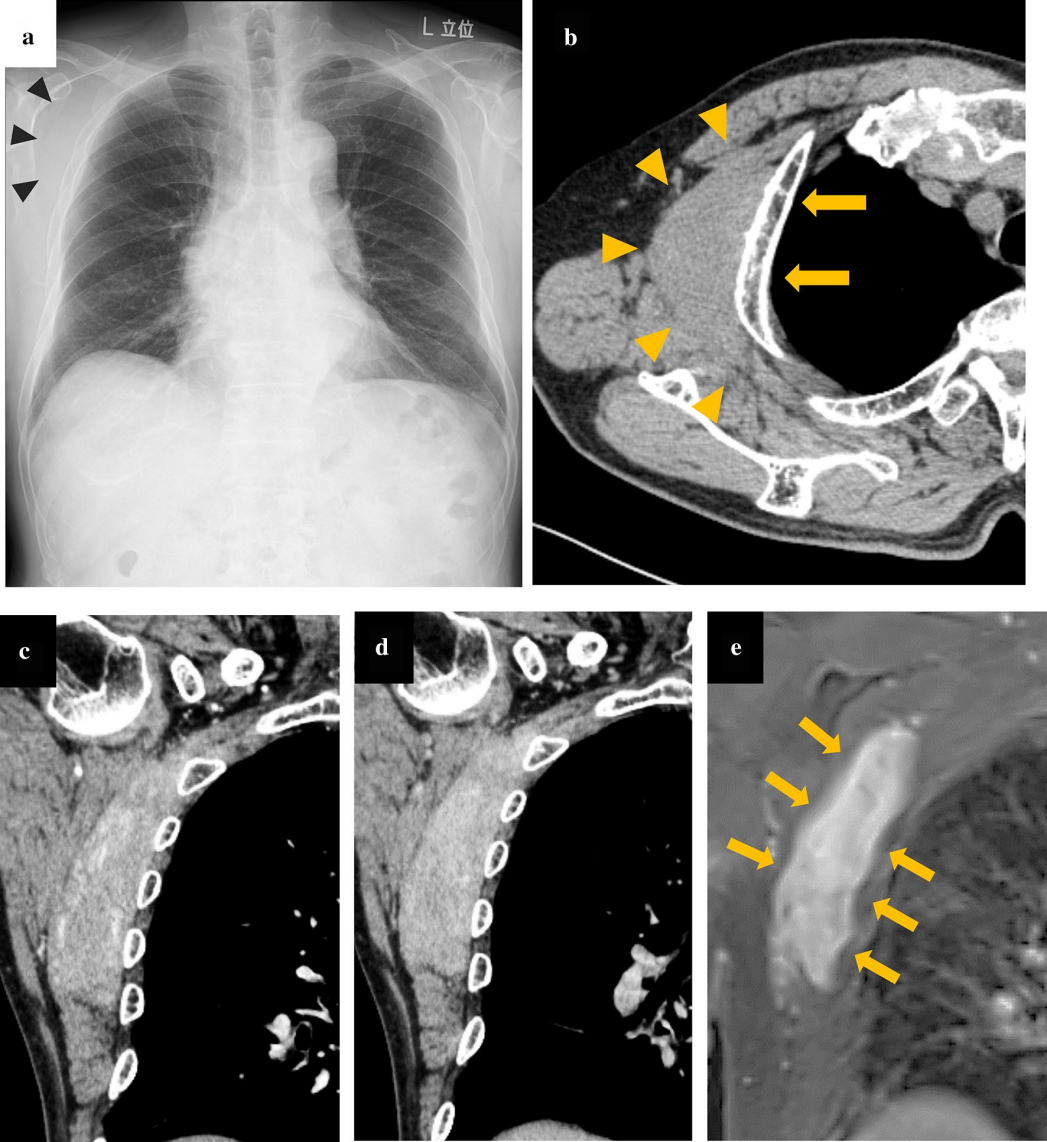
Radiological imaging of a case of desmoplastic fibroblastoma in the chest wall. **a** Chest radiograph demonstrating soft tissue thickening at the right lateral upper chest wall (arrowhead). **b** Axial view of a non-contrast computed tomography (CT) scan demonstrating the location of the tumor (arrowhead) and cortical bone erosion of the second rib (arrow). **c**, **d** Coronal view of a dynamic contrast-enhanced CT scan demonstrating the line of abundant vascularity at the periphery of the tumor during the early phase (**c**) and homogeneous enhancement during the delayed phase (**d**). **e** Coronal view of gadolinium-enhanced T1-weighted magnetic resonance imaging (MRI) with fat suppression, demonstrating inhomogeneous enhancement in the tumor, as well as rim-like contrast enhancement with uniform thickness at the tumor periphery (arrow)

A 79-year-old man was admitted to our hospital after presenting with right shoulder pain that had continued for a few months. On admission, physical examination revealed winging of the right scapula. A 4 × 4 × 2 cm mass had been detected at the right second rib on chest computed tomography (CT) scans two years prior. A chest radiograph revealed soft tissue thickening in the right lateral upper chest wall (Fig. 1a). Chest CT scans demonstrated a disk-shaped, well-defined, homogeneous mass with muscle-like density, measuring 12 × 10 × 4.5 cm in size (Fig. 1b). The mass extended from the axilla to the infrascapular region along the thoracic rib cage. CT scans in the bone window setting showed cortical bone erosion of the second rib. Dynamic contrast-enhanced CT scans revealed a region of abundant vascularity at the periphery of the tumor during the early phase (Fig. 1c) and homogenous enhancement during the delayed phase (Fig. 1d). The abundant vascularity branched from the lateral thoracic and thoracodorsal arteries. MRI revealed iso-intensity to muscle on T1-weighted images, slightly high-intensity to muscle on T2-weighted images, as well as heterogeneous enhancement in the tumor and rim-like contrast enhancement at the periphery, with uniform thickness on gadolinium-enhanced T1-weighted images with fat suppression (Fig. 1e: arrow). We strongly suspected the tumor to be desmoplastic fibroblastoma based on tumor localization and preoperative images.

**Fig. 2:**
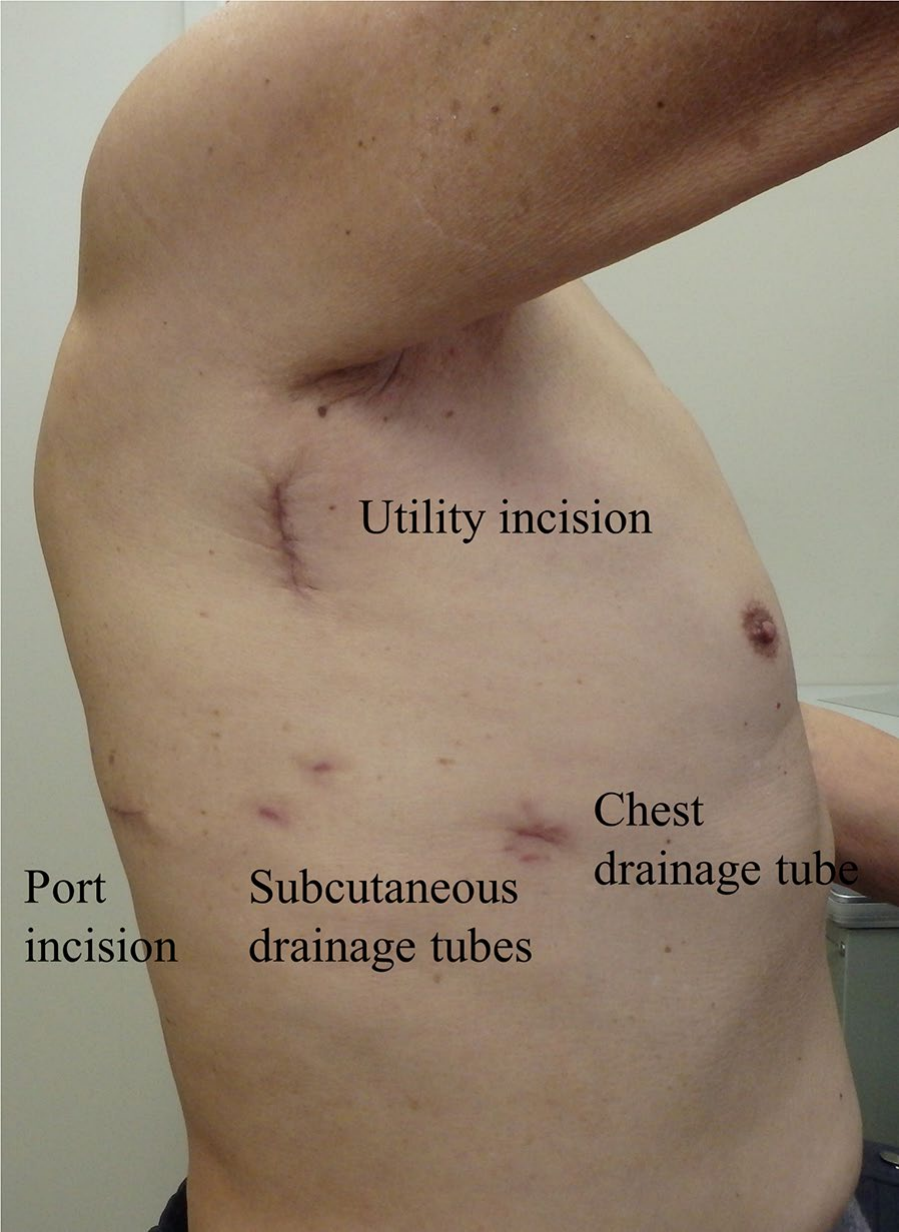
Marginal excision of the tumor through a small skin incision with videoscopic assistance

The patient refused a biopsy examination, preferring to undergo surgery through a minimal incision because of his shoulder pain. Marginal excision was performed through a small incision with the assistance of a thoracoscope (Endoeye®, Olympus, Tokyo, Japan). The tumor margin was marked on the skin surface with ink under ultrasound guidance. A 4 cm vertical utility incision was made along the middle axillary line, along with a 2 cm port incision at the triangle of auscultation (Fig. 2). A wound protector (Alexis®, Applied Medical, California, USA) was placed in the utility incision to retract the chest wall. The right scapula was retracted laterally using a retractor (Lobster Retractor System®, BOSS instruments, Virginia, USA). The tumor was excised with a surgical margin of approximately 3–5 mm using an energy device (HARMONIC HD 1000i shears®, Ethicon Endo-Surgery, Ohio, USA). The tumor had adhered strictly to the second rib. We considered that the tumor originated from serratus anterior muscle on the second rib, and then resected 5 cm of the second rib. The resected specimen was extracted through a small incision using a single-use polyurethane bag (Flexible Catcher ®, Japan Medicalnext, Tokyo, Japan), followed by the insertion of subcutaneous and chest drainage tubes. Frozen section diagnosis confirmed a benign fibroblastic tumor with negative surgical margins.

**Fig. 3:**
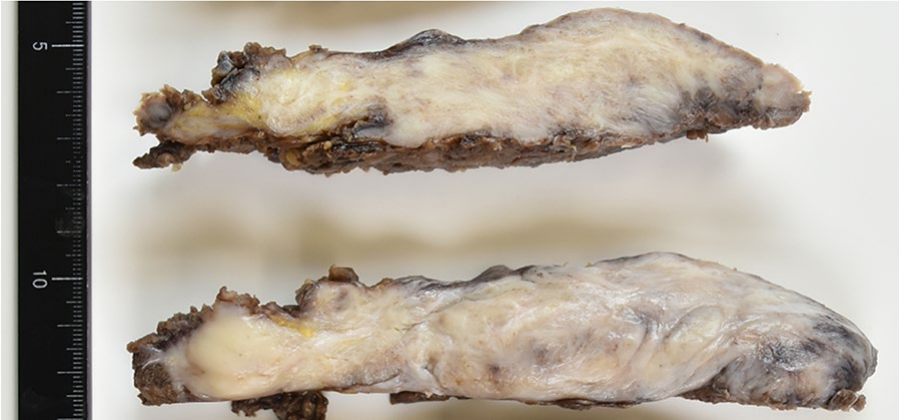
Macroscopic findings of the tumor. The cut surface of the resected tumor exhibiting a heterogeneous, colored appearance, with a gray layer visible at the periphery of the tumor

**Fig. 4:**
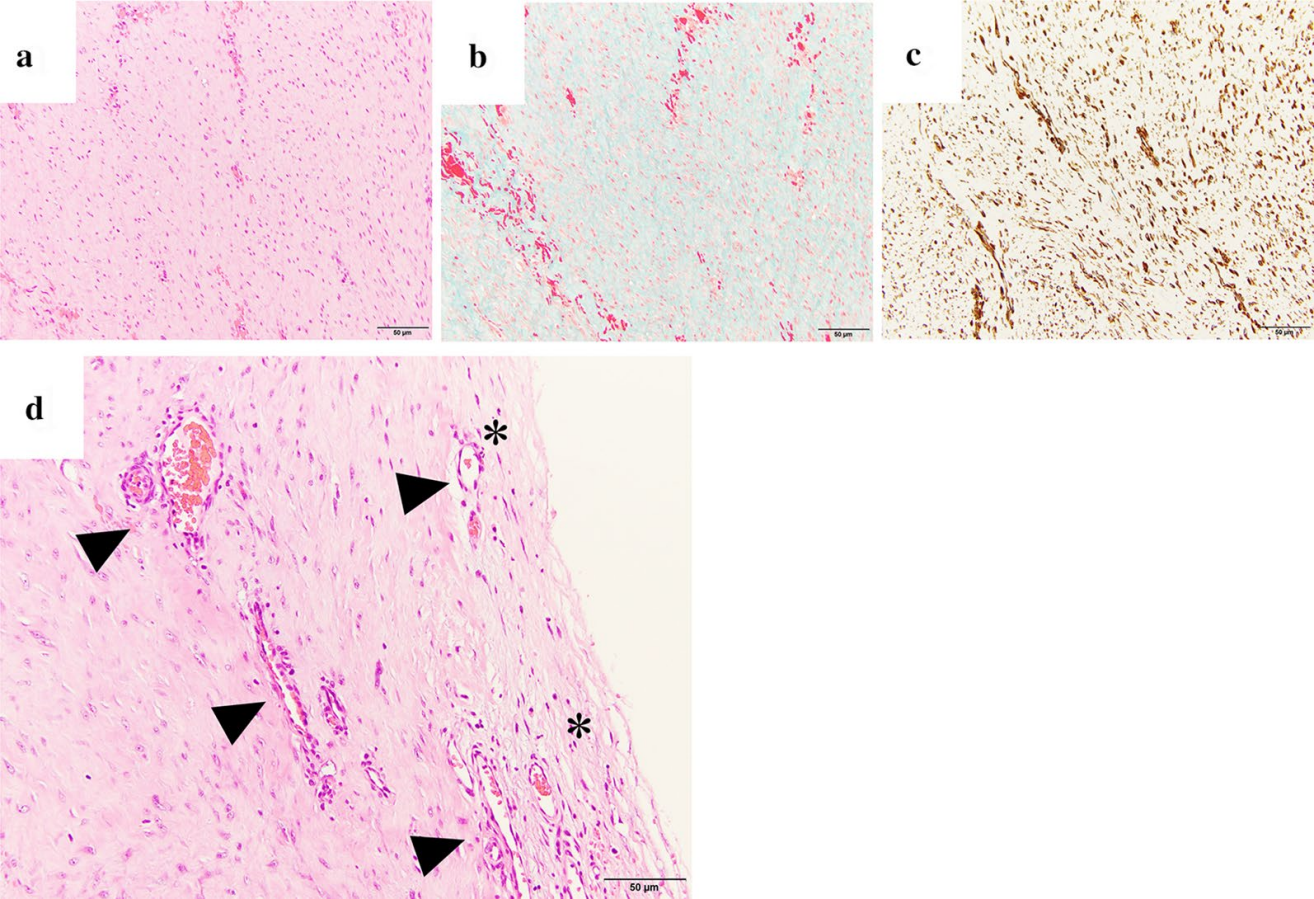
Histopathology of the tumor. **a**, **b** Desmoplastic fibroblastoma cells are stellate or spindle-shaped cells undergoing infrequent mitotic activity, embedded in an abundant collagenous stroma (**a** hematoxylin and eosin (H&E) staining, **b** elastica van Gieson staining). **c** Immunohistochemistry, demonstrating cells positively labeled for vimentin. **d **Examples of a capsule-like structure of fibrous tissue (*) and the abundant vascularity visible at the periphery of the tumor (arrowhead). (H&E staining)

The resected specimen was an elastic, hard, disk-sharped mass, measuring 13 × 12 × 5 cm in size. The cut surface had a homogeneous, white-colored appearance, with a gray layer visible at the periphery of the tumor (Fig. 3). Pathological examinations confirmed the presence of cells that were stellate or spindle-shaped, undergoing infrequent mitotic activity; these cells were embedded within an abundant collagenous stroma (Fig. 4a, b). Immunohistochemical analysis revealed that the tumor cells were positive for vimentin (Fig. 4c) and negative for α-smooth muscle actin, CD34, desmin, and β-catenin. The tumor was encapsulated by a thin layer of fibrous tissue and had infiltrated the adjacent skeletal muscle (Fig. 4d). A region of abundant vascularity was observed at the periphery of the tumor (Fig. 4d), which was consistent with an area of rim-like contrast enhancement on gadolinium-enhanced T1-weighted MR images with fat suppression (Fig. 1e). No neoplastic cells were identified at the surgical margins, and there was no bone invasion of the second rib. The tumor was diagnosed as a desmoplastic fibroblastoma. The patient’ s postoperative course was uneventful, and his shoulder pain was relieved after the surgery.

## Discussion 

An extremely rare case of desmoplastic fibroblastoma was reported. Desmoplastic fibroblastoma should be considered in the differential diagnosis when desmoid-type fibromatosis in the chest wall is clinically suspected. Rim-like contrast enhancement at the periphery of the tumor with uniform thickness on gadolinium-enhanced T1-weighted MR images with fat suppression is the unique imaging feature in desmoplastic fibroblastoma that can distinguish it from desmoid-type fibromatosis preoperatively. However, when performing marginal excision of desmoplastic fibroblastoma in the chest wall only by diagnostic imaging before surgery, as in our case, it is necessary to consider the treatment strategy in case of desmoid-type fibromatosis as a result of pathological diagnosis.

Desmoplastic fibroblastoma is a unique form of benign, fibrous, soft tissue tumor [1–4]. These tumors have been observed at a wide range of anatomical sites, although they most commonly arise in the lower limbs and feet, the back, and the upper extremities, including the shoulder, upper arm and forearm, and hand [1–4]. The lesions typically infiltrate the adjacent fat and skeletal muscle tissues, although they rarely involve the bone [1–3]. Desmoplastic fibroblastoma in the chest wall is quite rare, with only seven cases having been reported in the literature [9–15]. These tumors have the potential to grow aggressively, and two cases have involved bone invasion [13, 15].

The following fibrous tumors should be considered in the differential diagnosis of tumors occurring in the infrascapular region: desmoplastic fibroblastoma, desmoid-type fibromatosis, solitary fibrous tumor, and elastofibroma dorsi [9]. Among these tumor types, some of the imaging features of desmoplastic fibroblastoma are also common characteristics of desmoid-type fibromatosis, also called desmoid tumor [3–7, 11]. CT scans typically reveal a well-defined inhomogeneous mass with a muscle-like density [13–15], without calcification or the presence of cystic lesions [3]. MRI demonstrates findings of low- to iso-intensity to muscle on T1-weighted images, low- to slightly high-intensity or mixed-intensity on T2-weighted images, and heterogeneous enhancement on gadolinium-enhanced T1-weighted images with fat suppression [5–7]. Ultrasound reveals mixed echogenicity [5, 6], and positron emission tomography reveals the diffuse uptake of fluorine-18 fluorodeoxyglucose [7].

An imaging feature that can distinguish desmoplastic fibroblastoma from desmoid-type fibromatosis is rim enhancement [16, 17], which is characterized as rim-like contrast enhancement at the periphery of the tumor, with uniform thickness on gadolinium-enhanced T1-weighted MR images with fat suppression [16]. Rim enhancement is considered to represent the abundant vascularity of the outer capsule-like fibrous tissue relative to that inside of the tumor [5, 16]. However, the clinical utility of rim enhancement as an imaging feature remains limited, as certain characteristics are not fully understood [5–7]. For example, the capsule-like structure is a thin fibrous tissue, which appears dark and exhibits less enhancement on gadolinium-enhanced T1-weighted MR images [5–7], and abundant vascularity has been found inside of, but not around, the tumor by Doppler ultrasound [5].

We consider that rim enhancement can represent an area of infiltrative growth with abundant vascularity. A recent study reported the characteristics of the cells of a patient with a tumor that grew invasively into the surrounding tissue, which were distributed compactly at its periphery, forming a gray-colored layer [3]. The inside of the layer was lined with regions of abundant vascularity, while the outside was encapsulated with thin fibrous tissue. This layer could be superimposed onto the region showing rim enhancement on gadolinium-enhanced T1-weighted MR images with fat suppression. A capsule-like structure can result in a smooth and clear margin. The signal intensity of rim enhancement may vary among individual tumors due to their cellularity and vascularity. To the best of our knowledge, in the English-language literature, there are no descriptions of the vascular distribution of desmoplastic fibroblastoma on dynamic contrast-enhanced CT. Therefore, further studies are necessary to confirm our hypothesis.

Patients diagnosed with desmoplastic fibroblastoma exhibit favorable surgical outcomes after marginal excision [3, 4], although the tumor may be misdiagnosed as desmoid-type fibromatosis and treated with a wide resection [3]. Therefore, the correct preoperative diagnosis of the type of tumor is important for preventing overtreatment that may result in a loss of function [3, 5]; however, this remains challenging, and the exact diagnosis depends on pathological and immunohistochemical analyses [3–5, 11, 18]. Therefore, a well-planned incisional biopsy preceding a definitive excision is recommended when a mass in the chest wall exceeds 5 cm in diameter [8].

As an alternative to conventional open surgery, video-assisted surgery has been proposed for the resection of chest wall tumors, as it allows for a reduced incision size and less tissue trauma [19]. In the present case, 5-mm 30-degree video-thoracoscopy was useful for visualizing the surgical margins in the deep tissue. The tumor size was obviously larger than the incision size; nevertheless, the resected specimen was easily extracted through the small incision due to its high elasticity. The minimally invasive surgery facilitated the accelerated recovery of this patient, who was experiencing shoulder pain caused by tumor compression. Marginal excision of the tumor with videoscopic assistance through a small incision may be an effective alternative approach for the treatment of desmoplastic fibroblastoma in the chest wall.

When performing marginal excision only by preoperative imaging examinations, as in our case, it is necessary to consider the treatment strategy in case of desmoid-type fibromatosis as a result of pathological diagnosis. Marginal excision has insufficient surgical margin to reduce the local recurrence of desmoid-type fibromatosis [20–22]. However, additional resection of the upper chest wall may damage important structures such as the brachial plexus and subclavian arteries and veins [20–22]. Therefore, postoperative adjuvant radiation therapy has recently been recommended as an alternative to additional resection [20–22]. With these in mind, we had prepared two scenarios: first, if the frozen section diagnosis was suspected to be desmoid-type fibromatosis, additional resection of infiltrated skeletal muscles would be performed through a large incision. Second, if the postoperative pathological diagnosis was desmoid-type fibromatosis, postoperative adjuvant radiation therapy would be performed.

The histopathological features of desmoplastic fibroblastoma include the presence of bland stellate or spindle-shaped fibroblasts, as well as myofibroblasts undergoing infrequent mitosis that are embedded within an abundant and dense collagenous matrix, with low to moderate vascularity [1–4, 11, 18]. Immunohistochemical testing is useful for confirming the histopathological diagnosis [4], with tumor cells that are diffusely positive for vimentin, focally positive or negative for α-smooth muscle actin, and preferably negative for other markers (4, 18).

## Conclusion

Desmoplastic fibroblastoma in the chest wall is extremely rare, but should be considered in the differential diagnosis when desmoid-type fibromatosis is clinically suspected. Gadolinium-enhanced magnetic resonance imaging is helpful in confirming the differential diagnosis.

## Data Availability

The authors declare that all data in this study are available within the article.

## Abbreviations

CT: Computed tomography
MR: Magnetic resonance
MRI: Magnetic resonance imaging
